# Some Points in Lunacy Practice in Relation to the General Practitioner

**Published:** 1897-12

**Authors:** J. E. Shaw

**Affiliations:** Professor of Medicine, University College, Bristol; Physician to the Bristol Royal Infirmary


DECEMBER, 1897.

### SOME POINTS IN LUNACY PRACTICE IN RELATION TO THE GENERAL PRACTITIONER.

Abstract of the Presidential Address delivered on October 13th, 1897, at the Opening of the 24th Session of the Bristol Medico-Chirurgical Society.

BY

J. E. Shaw, M.B. Ed.,

*Professor of Medicine, University College, Bristol; Physician to the Bristol Royal Infirmary.*

After some preliminary remarks, the speaker proceeded to say that almost ever since he had been engaged in practice he had acted as medical adviser to the magistrates of the city in the consideration of cases supposed to be of unsound mind. With this and from experience gained in private practice, he had examined between three and four thousand persons as to their mental condition: this involved the consideration, not only of the question of sanity or insanity, but also what measure was best adapted in each case to best-ensure the welfare of the patient and the safety of the public—whether asylum, workhouse, single patient, or mere domestic care. Furthermore, since being a defendant in a law-suit some years ago, he had paid attention to Lunacy Law as it affects the interests of medical men. Under these circumstances he would crave the attention of his audience while as a practitioner of general medicine he discussed certain topics of lunacy procedure in which they, as non-specialist practitioners, also were more or less interested. About metaphysics, psychology, or even pathology, he would say nothing. The first subject for discussion he had headed

DESPECIALISATION, by which he designed to infer that the diagnosis of insanity was from the legal aspect no longer a special psychological or even medical function. This was shown (1) by the fact that previous to the Act of 1890 two medical certificates of insanity sufficed to constitute any one to be a person of unsound mind, while under the present Act the final judgment lies entirely with the magistrate to whom the certificates are submitted; (2) in the Bill before the House of Lords last session, upon the grounds that it is "expedient to extend the disqualifications for signing medical certificates," a clause was introduced debarring the medical superintendent or other licensee of a private asylum, or anyone in their employ, from signing a certificate or petition of insanity for their own or any other licensed house; (3) at the trial of a man for murder at Nottingham, before Mr. Justice Day, the prisoner was pronounced insane both by the gaol surgeon and medical superintendent of the Borough Asylum. Among other statements, the judge said "the only question was whether the prisoner was insane and did not know the nature and quality of his act. . . . He did not attach that value to medical evidence that some did: madness was easily detected by those who associated with the person affected; he should take the evidence of a prison warder, say, who had him in his charge as quite as valuable as that of the gaol surgeon . . . medical evidence was to be looked upon with great suspicion." The prisoner was found guilty. (4) At the last Wells Assizes a man was tried for the murder of his wife and child; medical evidence was given to the effect that prisoner was epileptic and committed the act in a state of automatism. The judge said "the question was whether prisoner knew he was killing the woman and child, knew the quality of the act, whether it was right or wrong. He had never heard that automatons, as some human beings were supposed to be, were relieved of their responsibility by law. It was not so, unless it was shown that they were incapable of distinguishing right from wrong." Jury found that the prisoner was insane. The judge adhered to the old noxious definitions of insanity and criminal responsibility directly in the face of medical evidence. The juries acted, however, quite independently of the judge's ruling in their diagnosis of insanity, accepting it in the one case and refusing it in the other. Above the Lord Chancellor, judges, Commissioners and Masters in Lunacy, psychologists and general practitioners, the British juryman is the ultimate judge of insanity, only the Crown apparently being able to set aside the decision of the jury.

### MEDICAL CERTIFICATES.

The diagnosis of insanity being regarded legally as dependent only upon the exercise of common sense, and not upon technical knowledge, the Act of 1890 directs that where two medical certificates are required for a case one must be given by the ordinary medical attendant of the patient. This imposes the responsibility of signing lunacy certificates upon every one engaged in practice. Some practitioners are averse to signing on account of dread of legal proceedings; but whatever faults the present Act may possess, it seems to be fairly effectual in protecting medical men, where they have acted with care and in good faith. The Lanchester case[1] forms a good precedent and valuable object-lesson on this point. The insertion in the present form of the words "at the time of examination" in the heading of "Facts observed by myself" often makes it difficult to certify persons whose insanity lies in their conduct rather than their intellect, as at the time of examination they may be behaving rationally. Comparatively little importance should be paid to the information afforded by others; it is often exaggerated unfairly or minimised unduly for private reasons. The evidence entered in the certificate may be painful, or even libellous. Dr. Rayner, in his address[2] at Carlisle, doubted whether it was not a breach of professional confidence to

[1] *Lancet*, 1895, ii. 1175; *Brit. M. J.*, 1895, ii. 1114, 1127.

[2] *Brit. M. J.*, 1896, ii. 797.

commit these facts to writing. This is probably not so. The certificate is analogous to the medical report in life insurance examination. It is designed to be seen by a limited number of official persons only—the examining magistrate, asylum superintendent, and Lunacy Commissioners. No possible opportunity should be given for any other person to see it. The Act gives no explicit direction as to what is to be done with the completed certificate, beyond that finally it has to be placed before the magistrate along with the petition and statement. Commonly, but improperly, it is handed over unenclosed to the friends, so that any one may read it or use it for improper purposes. It is almost certain that damages would be recoverable if it were proved to a judge and jury that such a course had been pursued; it would be held to constitute a want of reasonable care on the part of the medical man. The certificate is not drawn up to prove anything to the friends; they have no responsibility in its contents, nor power of utilising it in a legitimate way except by presenting it to the magistrate. The plan to be adopted is to enclose the completed certificate in a sealed envelope directed to the magistrate and endorsed "Medical Certificate respecting A. B." If any person other than the magistrate peruses this certificate, it is not through want of care on the part of the medical certifier.

### EXPENSIVE ASYLUMS.

Although in their special Report, issued last January, the Commissioners put forward reasons to show that there is no real increase of insanity in this country, yet the Report for 1896 just issued shows that the number of persons under certificates had increased during the year by 3000; in 38 years the proportion of insane in the population to each 10,000 persons has risen from 18 to 32; on the other hand, the number of patients in licensed houses grows less rather than greater. From this it follows that rapidly increasing accommodation must continue to be made in County and Borough Asylums if the plan at present in vogue in England for dealing with insane paupers is to be continued unaltered. The cost per bed of building and equipment of asylums may be estimated at about

£200: Claybury may be selected as an example of a pauper asylum of the best and most advanced character in England, and upon that building the entire expenditure was £579,000 for 2500 patients, or £238 per bed; at our own asylum at Fishponds tenders have been accepted for the addition of 150 beds at an aggregate cost of £45,000, or £300 per bed. Dr. Benham in his last report justly complains of the excess of incurable cases, mostly dements, which are thrust upon him yearly, and which accumulate in the asylum, their lives being prolonged by the care and good food they receive; these hopeless cases fill uselessly the asylum wards, as well as exert a deleterious influence on the acute and curable cases. For the purpose of treatment, the insane may be divided into four classes: (*a*) the *hospital* class, of very acute mental disorder; (*b*) the *asylum* class, with disorder less active, but requiring constant control; (*c*) the *colony* or employable class; and (*d*) the helpless or *workhouse* class. Without discussing either asylum management or the treatment of the insane, it may be said that all ratepayers are interested in the large and increasing sums of money spent upon asylum buildings. Is it necessary, or even beneficial, to a large proportion of these persons for them to be detained in these palatial edifices? Certainly the classes (*a*) and (*b*) must have all the full advantages of an asylum, with its hospital block for their proper care and treatment, and no diminution of expenditure can be demanded in respect of these two groups. Leaving, therefore, these classes out of consideration, is there no more economical and wholesome way by which classes (*c*) and (*d*) can be managed and maintained? For the third class, whose degree of self-control admits of their being trusted with partial liberty under supervision, why should they not be boarded out or located in colonies, and so maintained at a cost greatly less than in an asylum? What is there in English social life which makes it impossible to do in England what is done all over Scotland, where from 15 to 20 per cent. of the insane paupers are boarded out, and in Berlin, where the population is much denser than in any city in this country? Colonies of industrially occupied lunatics have been for centuries at Gheel, also at Lierneux in Belgium, at Alt-Scherbitz near Halle, in many places in France, and at Kankakee and other places in the United States. We have already in England industrial colonies for epileptics established: why should not the suitable members of the insane population be also thus healthfully employed in partly earning the cost of their maintenance? As for the fourth class, composed mainly of incurable dements, why should they have room provided for them in costly edifices like modern asylums? Either on the one hand they should be housed in what may be called workhouse asylums, erected at comparatively small cost, and without the expensive decorations and accessories considered necessary for the treatment of acute and curable cases; or, on the other hand, why should they not be detained in greater numbers in the provincial workhouses? The 4/- grant from Government in respect of asylum cases should be payable equally in respect of those certified imbeciles detained in a workhouse. For the many persons of very limited means, who are sent as private patients to public asylums, there should be separate wards or blocks for their reception at a cost of from one to two pounds a week, as the old mansion at Claybury is used. The educated and often refined private patients in most asylums are mingled with the paupers, and have to endure their coarse language and rude behaviour.

### UNCERTIFIED CASES

which present to practitioners a far larger class, and one more difficult to deal with, are those who (*a*) may not, and those who (*b*) can not, be certified from different reasons. Firstly, of those who, although fully insane, are not permitted to be certified. The ratio of private patients in this country is diminishing; this is owing partly to the publicity due to the necessary cognizance of the case by a magistrate, and partly to the dislike to asylums and certification by the public. Dr. Savage stated (in a deputation to the Commissioners) that the public exclaim "Anything but certification," when it is proposed to place a patient under certificates. There is no doubt that from aversion to certification on the part of the friends many insane persons are kept in private care with very inadequate treatment. Secondly, of those who cannot be certified in the existing state of the law there are many kinds, who may be formed into one or two groups. The confirmed inebriates form a large and important section practically undealt with, the well-intentioned Acts of 1879 and 1888 being inoperative to all intents and purposes. The just issued Report of the Inspector states that there are twelve Retreats in this country, with 110 patients on January 1st, 1896; not so many under treatment in the whole country as probably could be found to exist in many a single parish. One cause of this failure is that the applicant for admission has to go before two magistrates to sign a declaration that he is entering the Retreat of his own free will; the other more deplorable cause is that, unable to control himself, he cannot bring himself to take the step; he is compelled by law to be the sole person who can bring about his own enforced deprivation from stimulants, all others being prevented from taking active steps to save him. The medical superintendent of every Retreat does but reiterate in the Report his indignation at this solemn and hideous mockery. Another class of anxious cases consists of those who are mad medically but not legally. There are many men who by extravagance, dissipation, flagrant immorality and disregard of social conventionality have ruined their families and friends; there are, again, many women who by secret drinking and drugging, by hysterical possession, by what we must call kleptomania, and other moral delinquencies, have brought equal misery into their homes: the families and medical attendants can do nothing until the actions of these persons have become detrimental to society at large. The law protects society from the delinquent, but not the delinquent from himself. In the Code of Civil Law which will come into operation in Germany in the year 1900 it is specifically provided: "Under guardianship may be placed those who (1) by prodigality, or (2) by drunkenness, expose themselves or families to the dangers of poverty or distress, or endanger the safety of others." Would that there were some such legal provision in our own country! Besides these, there are many other cases of neurasthenia, epilepsy, hystero-epilepsy, erotomania, &c., which cannot be admitted to asylums because they are not legally certifiable; there are no wards in ordinary hospitals where they can be compulsorily detained, so they are left to be a perpetual source of misery both to their friends and to themselves.

Have no means been proposed by which society should rid itself of these burdens? A recent issue of the *Journal of the American Medical Association* has a long argument in support of its proposition to kill all idiots. Urquhart at Montreal said that in Scotland in former days all male epileptics were castrated, in order to prevent the propagation of the heredity; Sir J. Crichton Browne quotes the advice of the New York Medico-Legal Society to the effect that suicide should be recommended to those more or less insane. These methods, no doubt, are simple and effective! But if hopeless cases of insanity are consigned to the lethal chamber the same process would be easily extended to other classes of the community, and there is no knowing where the application of the principle might stop. Such suggestions will find no supporters in this Society. Only the present state of the law in England prevents that from being done in this country which is done in Scotland in cases legally uncertifiable, as well as in cases also of distinct but transient insanity where it is not desired to place the patient in an asylum. An order is signed by a near relative, and a certificate by one medical man; by this conjoint means the patient is sent to a private house named in the order, and is there and thereby detained in the first instance for six months, which can be subsequently extended. With this arrangement there is no publicity, no magisterial interview or investigation, no subsequent slur upon the patient for having been an inmate of an asylum, while occasional visits of Commissioners guarantee that the patient is properly cared for. That the plan is not abused is shown by the fact that there has been no action for illegal detention in Scotland for more than twenty years. Were this process in force in England, how much misery could be prevented! A joint Committee of the British Medical Association and the Medico-Psychological Association was appointed to consider the question, and draw up a code of rules which, if enacted, would make this possible. The interview of this Committee with the Lunacy Commissioners is reported in the issue of the *British Medical Journal* for June 5th, and their proposed clauses to be added to the Lunacy Amendment Bill before Parliament last Session were printed in the *Journal* for July 24th. The Bill was dropped, but no doubt will be re-introduced next Session. As already stated, there is no place in wards of ordinary hospitals where these non-certified cases can be detained; but if they become legally certifiable for temporary detention, as above proposed, some definite accommodation will have to be provided. Is it Utopian to expect that in each large town there should be a hospital for nervous diseases? Here could be brought the simple melancholic who requires feeding, the neurasthenic who requires massage and isolation, the hystero-epileptic who requires occasional restraint, the simple epileptic might be taken thither when attacked in a public place, thither also might go the tippler or confirmed inebriate for whom a six months' detention is not sufficiently long, and there also many cases of insanity as yet uncertifiable (such as general paralysis in early stage), might be detained and deprived of the power of harming themselves or others, until such time as they could be fully certified and transferred to an asylum. Furthermore, there should be a separate block whither severe cases of acute insanity could be taken as a receiving ward. Cases of acute delirious mania, delirium tremens, post-epileptic mania, and desperately suicidal melancholia are constantly occurring, and in their violent and excited condition have to be removed to the County or Borough Asylum, possibly many miles away. Why should they not be taken to the receiving ward of this hospital for nervous diseases, and kept there until, the extremest violence having passed off, they could with less danger be transferred, if still necessary, to the distant asylum? Were not these cases compelled to travel when unfit in body and mind so to do, how many fewer would be found to have their ribs broken when they die a few days after admission to the asylum! Payment should be demanded from patients in this hospital according to their means; and how best it might be provided with a medical staff is a matter for consideration, but the insane block should undoubtedly be under the direction of a physician who had held an asylum appointment.

